# Case of Poisoning by Datura Stramonium

**Published:** 1834

**Authors:** B. F. Williams

**Affiliations:** Cincinnati


### IX.—Case of Poisoning by Datura Stramonium.

*Communicated by Dr. B. F. Williams.*

In the summer of the year 1832, about 9 o'clock P. M., I received a call to visit Ann Maria O. æt. between 6 and 7

years, of a delicate nervous constitution, hereditarily predisposed both to phthisis and epilepsy; of the latter of which diseases she had suffered several violent attacks, which usually seemed to be brought on by gastric or gastro-enteritic irritation, rather than any original morbid cerebral action, for in common the exhibition of an emetic or an emetico-cathartic, speedily allayed the paroxysms and restored the various functions.

On arriving, I found Dr. A., my former Preceptor, there, who had administered an emetic of Ipecacuanha, on the supposition that she was laboring under an attack of epilepsy "her old complaint."

She had vomited little—appeared much agitated—seemed somewhat delirious—stared and looked wildly—muttered incoherently—her superior extremities were in constant and rapid motion, as were the inferior, though less so. Upon raising her into a setting position, she permitted her head to fall back, threw out her arms and endeavored to lay hold of something to prevent her from falling backwards, and seemed vertiginous as one who had been whirling rapidly on his feet.

A feather was thrust into the throat to tickle the fauces and assist the action of the emetic, but nothing was discovered in the ejected matter, (which was some warm water and the Ipecacuanha which she had taken,) that could have given rise to such distressing symptoms.

Upon further inquiry it was ascertained that about six o'clock P. M., she came from play—complained of weariness—lassitude—wished to sleep—felt slight nausea—said it was "so dark she could not see well"—rubbed her eyes with her hand—complained of pain in her forehead—and had some slight muscular tremors in her upper and lower limbs and face.

Upon opening the eyelids, the pupils were discovered enormously dilated—the iris being drawn to the ciliary ligament so closely as to measure less than half a line in width. A bright lamp was then brought near the eye and so placed as to fall directly upon the axis of each, yet the glaring vividness of the light produced not the slightest effect upon the retina, the pupil remaining rigidly dilated, and totally insensible to its natural stimulus.

It was then suggested that the patient might perhaps inadvertantly have eaten of some of the noxious plants known to grow spontaneously on the commons throughout the neighborhood, and the symptoms were highly favorable to such a supposition. Nothing satisfactory upon this point could, however, be collected. She was ordered an infusion of green tea as a drink—a pediluvium—draughts to the feet—and cold water occasionally applied to the head, through the night.

Morning, 6 o'clock.—She was costive—had passed a restless night—no palliating effects from the remedies—muscular motions much increased—eyes wide open—wild—remaining totally insensible to light. In addition, it was discovered that she was also perfectly insensible to sounds, as several attempts were made to rouse and arrest her attention by this means but failed. She was constantly tossing her arms from side to side—sometimes appearing to tie and untie her bonnet, and to take it off—to hold up a book and read and spell—then, as if threading a needle, or catching at something in the air—picking berries and eating them—throwing sticks, and then ceasing suddenly all muscular movement for a moment—then calling aloud—whispering, laughing, crying, and talking to her playmates, all in the same breath.

Learned this morning, that she had, while in company with several other children yesterday-afternoon, amused herself with the young capsules of the Stramonium, and had chewed some of the green seeds; but whether she had swallowed any, could not be ascertained.

That the case was one of poison by the article named, was now beyond a doubt. But as she had already taken an emetic, in the commencement of the attack, without any particular or manifest advantage, it was deemed inexpedient to have recourse to that remedy again;—for, 1st. there was nothing, perhaps, to be gained by evacuating the stomach at present, and 2nd. there was a doubt whether, under the cephalic excitement, the stomach would be sensible to the impression of an emetic, and 3d. if the emetic should succeed, its prostrating effects might, under present circumstances, prove greater than the vital powers could sustain.

It was now proposed to employ a cold bath composed of vinegar and water, several times through the day, as an antispasmodic, which, together with its admitted antidotal effects in cases of vegetable poisoning, seemed to point it out as an agent peculiarly serviceable and appropriate in the present case,—and to administer a tablespoonful of a strong infusion of coffee alternately with the same quantity of sweetened vinegar and water every half hour. For the bath, however, mere sponging with vinegar and water, once an hour, was substituted.

4 o'clock, P. M., symptoms unabated—no evacuations from the bowels—no nausea—same prescription continued.

9, P. M., seemed less violent—has occasionally short intervals of repose—wakes more violent, but soon relapses to her former state. I remained during the night. About 2 o'clock in the morning had an hour of tranquil sleep—waked but seemed less violent and delirious than before—this was followed in two hours by a profound sleep almost approaching coma—waked in about an hour and a half—spoke, but we could not understand what she desired.

9, A. M. had fallen asleep soon after my departure, and not yet awaked—seemed quiet, save an occasional start and movement of the lips as if talking to herself.

12, M. had waked at 11, perfectly rational—pupils contracted to their natural size—could hear distinctly—said she had slept long—quite unconscious of what had passed—desired food.

From herself we now learn that she had chewed some of the seeds of the Stramonium, but had swallowed none.

She was ordered the following cathartic—

℞ Rhei. pulv. gr. iij.
Ol. Ricini. ʒii. mix.

repeated every two hours, till an operation was obtained—and a diet of rice—toast—boiled milk—and soup of chicken, for a few days.

*Cincinnati, June*, 1834.

